# Key digital health literacy competencies for citizens – A Delphi study

**DOI:** 10.1177/20552076251357375

**Published:** 2025-07-03

**Authors:** Mika Uitto, Merja Hoffrèn-Mikkola, Leena Paakkari

**Affiliations:** 1 7295 Seinäjoki University of Applied Sciences, Seinäjoki, Finland; 2 541605 Faculty of Sports and Health Sciences, University of Jyväskylä, Jyväskylä, Finland

**Keywords:** Citizens, digital health literacy, competencies, digital transformation, healthcare, health promotion, digital health

## Abstract

**Objective:**

Digital technologies provide citizens with many opportunities to increase control over and improve health, but their use requires digital health literacy. This study aimed to identify the most important competencies that citizens will need over the next 3 years to promote and maintain good health and wellbeing through digital technology.

**Methods:**

Using a three-round Delphi method, Finnish experts on digital health (*n* = 29) listed competencies needed by citizens over the next 3 years to promote and maintain their health and wellbeing through digital technology and then evaluated and ranked them based on their perceived importance. Qualitative and quantitative methods were used to analyse the data.

**Results:**

A broad list of competencies was produced, and the 17 most important were identified. The two most important competencies were the ability to use electronic identification and general IT proficiency.

**Conclusion:**

The findings suggest that being digitally health literate in the modern world goes beyond the ability to seek, assess, understand, and apply online health information. It also encompasses electronic identification, general IT proficiency, navigating and using digital health services, accessing personal health records, and ensuring online privacy and security. These results offer important insights into the competencies needed to meet current digital health challenges, with implications for both assessment tools and intervention strategies.

## Introduction

Digital transformation impacts all aspects of life, including health.^1^ Technological advances are driving change, not only directly through their applications in healthcare and health promotion, but also indirectly through their influence on the social, commercial, and environmental determinants of health.^1^ Healthcare systems must struggle with demographic changes, particularly ageing populations and the rising prevalence of chronic diseases.^2^ Digital health technologies have been suggested as one potential solution, offering new cost-efficient, high-quality, and personalized opportunities to promote the health and wellbeing of citizens.^3^ However, they may also introduce new demands on citizens’ competencies.^1^

Digital transformation efforts are advancing globally. By the end of 2023, all European Union member states had introduced some form of national or regional personal health records, though gaps in population coverage persist.^4^ The Union aims to bridge these gaps by ensuring that 100% of citizens have online access to their personal health records by 2030,^5^ and by enabling citizens to access their health records securely and easily across member states.^6^ Digital readiness among citizens supports these goals: 93% of EU citizens have used the internet^7^ and 55% report using it to search for health-related information, although usage rates vary significantly – from 80% in Finland to 36% in Bulgaria.^8^ On a global scale, disparities in digital health maturity are even more pronounced. While some countries have implemented comprehensive strategies and built advanced digital health systems, others are still laying the foundations for basic infrastructure.^9,10^ The World Health Organization^11^ classifies the use of digital health technologies into four categories: *technologies for healthcare providers*, *health system managers*, *data services*, and *citizens* (clients). This study focused on digital health technologies for citizens.

Digital health technologies are increasingly regarded as tools that empower citizens to actively manage their daily health both in healthcare settings and everyday life,^12,13^ marking a shift from the traditional role of patients as passive recipients of information and care.^14^ Their potential advantages include improved access to health information and services, online appointment booking, remote consultations, improved communication with professionals, as well as enhanced self-monitoring and health awareness enabled by wearable technologies.^15,16^ Despite their potential to enhance health outcomes and improve access to services, the benefits of these technologies remain unevenly distributed.^12,17^ Populations particularly vulnerable in the context of digital health include older adults, migrants, individuals with poor health, and those with low socioeconomic status.^18,19^ For these groups, the barriers to using digital health information and services may arise from insufficient digital or local language skills, lack of support or training, poor health, missing electronic identification or devices, and security and trust concerns.^18^

Disparate knowledge and skills (i.e. competencies) in using digital technologies constitute a key factor in digital inequities.^20^ A commonly used term to describe these competencies in the context of digital health is *digital health literacy*, a concept that has continued to evolve over time.^21,22^ It was originally defined by Norman and Skinner^23^ as ‘the ability to seek, find, understand, and appraise health information from electronic sources and apply the knowledge gained to addressing or solving a health problem’. Their Lily Model conceptualizes digital health literacy as comprising six core literacies: health literacy, traditional literacy and numeracy, computer literacy, media literacy, science literacy, and information literacy.^23^ Building upon this foundational model, subsequent research has largely focused on individuals’ abilities to find, evaluate, understand, and use health information obtained from digital sources.^21^ However, this approach may be too narrow to capture the full range of knowledge and skills required by individuals who are increasingly expected to manage and maintain their health using a diverse array of digital tools.^24,25^ Given rapid technological advancements, it is crucial to continuously update and refine our understanding of digital health literacy to reflect the competencies citizens require.^26–28^ In this study, we understand digital health literacy *as a set of knowledge and skills that enable citizens to promote and maintain good health and wellbeing through digital technology.*

Digital health literacy is a key determinant of health that can be enhanced through a range of interventions.^12,15^ However, ensuring their effectiveness requires a clear understanding of the essential competencies citizens need. Such knowledge can guide the development of digital health literacy to meet current challenges and support the design of improved interventions and assessment tools. This Delphi study aimed to answer the research question: *What are the most important competencies that citizens will need over the next three years to promote and maintain good health and wellbeing through digital technology?*

## Methodology

A three-round Delphi study was conducted, incorporating both qualitative and quantitative methodologies. The Delphi method was selected due to its capability for swift and anonymous data gathering and consensus generation on topics where scientific knowledge is limited.^29–31^ In this study, three Delphi rounds were conducted on a group of Finnish experts on digital health over a 9-week period between September and November 2024.

### Participants

A purposeful sampling approach and procedure by Okoli and Pawlowski^32^ was employed to build the panel of Finnish experts for this study. First, the research team identified the disciplines or skills relevant to the research question. These disciplines were health literacy, health promotion, wellbeing technologies, health communication, and digital healthcare. To identify potential experts in these fields, the researchers compiled a pool of candidates by extracting names from relevant publications or the websites of relevant organisations. The inclusion criteria for selecting experts were expertise in one or more of the identified disciplines (demonstrated through relevant publications or professional roles), proficiency in Finnish, and willingness to participate in the study. Next, the researchers discussed and ranked the qualifications of the experts to select the final expert panel for invitation to the Delphi study. The team contacted these potential experts via email and phone calls, inquiring about their interest in participating in the study. Willingness to participate in the study was confirmed either through written consent via email or verbal agreement over the phone. In case of unavailability, the experts were asked to suggest additional experts with similar skill sets. In total, 31 experts accepted the invitation, 29 completed Round 1, 26 completed Round 2, and 23 completed Round 3 (Table 1).

**Table 1.** Participant demographics.

	Round 1 (*n* = 29)	Round 2 (*n* = 26)	Round 3 (*n* = 23)
Gender			
Male	4 (13.8%)	5 (19.2%)	3 (13.0%)
Female	25 (86.2%)	21 (80.8%)	20 (87.0%)
Age			
30–39	4 (13.8%)	4 (15.4%)	3 (13.1%)
40–49	8 (27.6%)	7 (26.9%)	5 (21.7%)
50–59	13 (44.8%)	10 (38.5%)	12 (52.2%)
Over 60	4 (13.8%)	5 (19.2%)	3 (13.0%
Education			
Master's or equivalent level	11 (37.9%)	8 (30.8%)	10 (43.5%)
Doctoral or equivalent level	18 (62.1%)	17 (65.4%)	13 (56.5%)
Other, not specified	0	1 (3.8%)	0
Field of occupation			
Education	1 (3.4%)	1 (3.8%)	1 (4.3%)
Social sciences, journalism, and information	8 (27.6%)	8 (30.8%)	5 (21.7%)
Business, administration, and law	1 (3.4%)	2 (7.7%)	1 (4.3%)
Natural sciences, mathematics, and statistics	1 (3.4%)	0	0
Information and Communication Technologies (ICT)	2 (6.9%)	2 (7.7%)	2 (8.7%)
Engineering, manufacturing, and construction	2 (6.9%)	1 (3.8%)	1 (4.3%)
Health and welfare	14 (48.3%)	12 (46.2%)	13 (56%)
Occupation			
Research	8 (27.6%)	9 (34.6%)	6 (26.1%)
Education	9 (31.0%)	8 (30.8%)	7 (30.4%)
Social or healthcare professional	1 (3.4%)	1 (3.8%)	1 (4.3%
Entrepreneur	1 (3.4%)	0	0
Expert or development work	8 (27.6%)	7 (26.9%)	8 (34.8%)
Management	1 (3.4%)	1 (3.8%)	1 (4.3%)
Other	1 (3.4%)	0	0

The data were collected using three digital questionnaires (Webropol), with links emailed to the experts at each round. In all three questionnaire rounds, participants were required to consent to the use of their data as outlined in the attached privacy notice and to confirm their willingness to participate. Demographic questions were included in each round. The experts could participate in any round, regardless of whether or not they had taken part in the previous rounds. Before being sent to the experts, the questionnaires were pilot-tested with two individuals who were neither part of the research team nor experts on the topic.

### Delphi procedure

Round 1. The aim in this round was to encourage the experts to freely share their insights on the research phenomenon and to generate a list of items for the second questionnaire.^33^ The participants were asked the open-ended question, ‘Consider the following three years. What kind of digital health literacy will be important for citizens to promote and maintain good health and wellbeing for themselves and those around them?’ The respondents were requested to list as many competencies as possible and describe them in a few words. In the questionnaire, digital health literacy was defined as ‘knowledge and skills that enable citizens to promote and maintain good health and wellbeing through digital technology’. The responses of the experts were thoroughly read and discussed critically by all the authors. Overlapping mentions or duplicates were removed, and terminology was unified if necessary. The aim was to remain faithful to the data while recognising and articulating the statements as clearly as possible.^33^ This set of competencies was used to build a list of items for the second Delphi round.

Round 2. In this round, the experts were asked to rate the importance of each competency identified in the first round on a 7-point Likert scale. The items were presented in a randomised order. The Likert scale ranged from 1 = *not at all important* to 7 = *extremely important*. The responses of the experts were analysed quantitatively, and the items identified as most important were used for the final questionnaire round. Hence, the sums, means, modes, medians, standard deviations, and *Z*-scores were examined for each item. Additionally, agreement percentages were calculated by examining the percentage of experts rating an item with a score of at least 5, at least 6, and exactly 7. According to Diamond et al.,^34^ there is no gold standard for determining the point of consensus in Delphi studies. In the present study, we selected items with medians and modes of ≥ 5 and a *Z*-score of ≥ 0.5 for the final round. This cut-off point was based on the need to gather a manageable number of highly rated items for the final round. A less restrictive cut-off would have resulted in too many items, while a stricter cut-off would have overly reduced the item pool. The chosen cut-off was, nevertheless, somewhat arbitrary. As noted by Löfmark and Mårtensson,^35^ there are no universally accepted guidelines for cut-offs in the literature.

**Figure 1.**
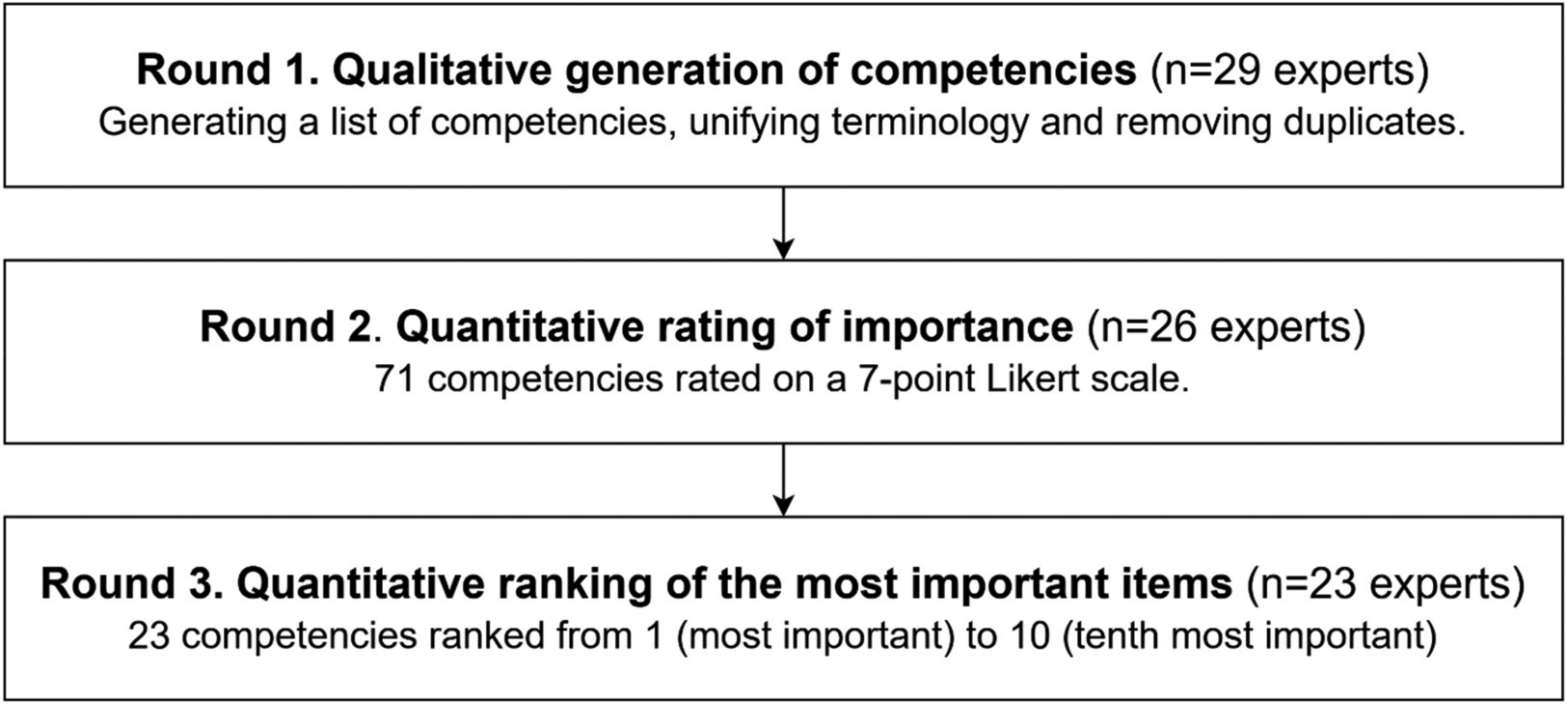
Flowchart of the Delphi process.

Round 3. In this round, the most important competencies based on the quantitative analysis from Round 2 were presented to the experts. From these items, the experts were asked to select the 10 most important for citizens over the next 3 years and rate them from 1 (most important) to 10 (10th most important). The most important item received 10 points and the tenth received 1 point. The sum scores, *Z*-scores, were again calculated for each item. Agreement percentages were calculated by examining the percentage of experts granting the items one point, five or more points, and ten points. To identify the most important competencies for citizens over the next 3 years to promote and maintain good health and wellbeing through digital technology, a *Z*-score of ≥ −0.58 was used as a cut-off value. Once again, this was an arbitrary value^35^; was applied for practical reasons. Figure 1 presents an overview of the three-round Delphi process.

## Results

### Round 1

In Round 1, 29 experts participated (response rate 93.5%). The experts produced a list of 300 competencies for citizens over the next 3 years. After removing duplicates and unifying the terminology, a total of 71 competencies were identified and used to form the list of items for Round 2.

### Round 2

In Round 2, 26 experts participated (response rate 83.4%). In this round, they were asked to rate the importance of each competency for citizens over the next 3 years on a 7-point Likert scale (see the ‘Delphi procedure’ section). All the identified competencies received relatively high ratings, with 60 out of 71 items achieving a mean score of at least 5.00. The *Z*-scores of the items ranged from −1.40 to 1.96. The selected cut-off (items with medians and modes ≥ 5 and *Z*-score ≥ 0.5) yielded 23 competencies. These were used as the items for Round 3.

### Round 3

In Round 3, 23 experts participated (response rate 74.0%). The experts were asked to select and rank the 10 most important competencies for citizens over the next 3 years (see the ‘Delphi procedure’ section). The most important item received 10 points, and the tenth most important received 1 point. The theoretical maximum sum score for each item was therefore 230 points. To identify the most important competencies, sum scores, *Z*-scores, and agreement percentages were calculated. The experts’ responses varied across the items from a sum score of 11 to 144, while for the *Z*-score, the range was −1.27 to 2.57. All 23 items received at least one mention in the experts’ top 10 lists. The selected cut-off (*Z*-score −0.58) was taken as yielding the 17 most important competencies for citizens. The competency rated as most important for citizens over the next 3 years was *the ability to use electronic identification* (sum: 144, *Z*-score: 2.57). The second most important was *proficiency in general IT skills* (e.g. using a computer, smartphone, and the internet) (sum: 115, *Z*-score: 1.73). All the 17 competencies identified as belonging to the most important are listed in bold in Table 2.

**Table 2.** The most important competencies for citizens over the next 3 years to promote and maintain good health and wellbeing through digital technology, as identified by an expert panel.

Competencies	Round 2				Round 3					
	Mode	Median	*Z*-score	% = 7	Sum	Mean	*Z*-score	% = 10	>= 5	% = 1
**1. Ability to use electronic identification.**	7	7	1.96	61.54	144	6.86	**2.57**	19,05	76.19	4.76
**2. Proficiency in general IT skills (e.g. using a computer, smartphone, and the internet).**	7	7	1.46	53.85	115	7.19	**1.73**	37,50	68.75	6.25
**3. Ability to find relevant contact information online and contact healthcare professionals digitally.**	7	6	0.82	38.46	107	6.29	**1.50**	11,76	64.71	5.88
**4. Ability to find relevant digital healthcare services.**	6	6	1.39	38.46	91	5.35	**1.04**	11,76	58.82	23.53
**5. Ability to apply personal health information from personal health records (e.g. MyKanta) for daily health-related activities and decision-making.**	6	6	0.60	26.92	82	5.13	**0.78**	0,00	50.00	6.25
**6. Ability to evaluate the reliability of digital information and sources.**	7	6	1.25	42.31	79	6.58	**0.69**	8,33	75.00	0.00
**7. Ability to apply health-related information from digital sources and make informed decisions in daily life.**	5	6	0.60	30.77	73	6.08	**0.52**	16,67	66.67	8.33
**8. Understanding and knowing how to apply security practices to protect privacy (one's own and that of others).**	7	6	1.39	42.31	60	6.00	**0.14**	10,00	60.00	10.00
**9. Ability to make and manage healthcare appointments electronically.**	7	6	0.74	34.62	58	5.27	**0.09**	9,09	72.73	18.18
**10. Ability to access and view personal health information in personal health records (e.g. MyKanta).**	7	6	1.03	38.46	48	4.80	**−0.20**	0,00	50.00	0.00
**11. Ability to share and manage personal information safely across various online platforms.**	7	7	1.75	65.38	46	5.75	**−0.26**	25,00	75.00	0.00
**12. Ability to provide informed consent for the digital processing and storage of personal data.**	7	6	1.03	38.46	46	5.75	**−0.26**	12,50	75.00	12.50
**13. Ability to fill out electronic applications related to health and wellbeing (e.g. for support applications and service applications).**	6	6	0.96	30.77	45	5.63	**−0.29**	0,00	75.00	12.50
**14. Ability to view, renew, and utilise electronic prescriptions.**	6	6	0.74	30.77	43	5.38	**−0.35**	0,00	62.50	0.00
**15. Ability to use digital information sources and search systems to find information.**	6	6	0.67	30.77	40	5.00	**−0.43**	0,00	75.00	0.00
**16. Ability to use digital citizen portals and their features (e.g. Suomi.fi).**	6	6	0.82	23.08	38	4.75	**−0.49**	0,00	50.00	0.00
**17. Ability to critically evaluate health discussions on social media.**	6	6	0.96	26.92	35	4.38	**−0.58**	12,50	25.00	0.00
18. Knowing one's rights and responsibilities in digital services and environments.	6	6	1.17	34.62	24	4.80	−0.90	0,00	60.00	20.00
19. Ability to interpret personal health information from personal health records (e.g. MyKanta).	5	6	0.74	30.77	22	4.40	−0.95	0,00	40.00	20.00
20. Ability to express thoughts and ask questions clearly in digital interactions and services.	6	6	0.53	23.08	22	3.14	−0.95	0,00	14.29	28.57
21. Ability to manage passwords required for logging in.	7	6	0.89	42.31	19	3.80	−1.04	0,00	40.00	40.00
22. Knowing where to find information about social and healthcare services online.	7	6	0.60	38.46	17	2.83	−1.10	0,00	33.33	33.33
23. Ability to act responsibly and respectfully online.	7	6	0.89	42.31	11	2.75	−1.27	0,00	25.00	25.00

The 17 highest-rated items from Round 3 are printed in bold.

## Discussion

This study aimed to identify the most important competencies that citizens will need over the next 3 years to promote and maintain good health and wellbeing through digital technology. Using a three-round Delphi method, Finnish experts produced a comprehensive list of competencies, of which 17 were identified as the most important.

According to the experts, the most important competency for citizens was the *ability to use electronic identification*. This was followed by *general IT proficiency*, such as the ability to use computers, smartphones, and the internet. These can be seen as prerequisites for securing access to most of the digital social and healthcare services available to citizens. Lack of electronic identification is a major barrier to digital health use, especially affecting vulnerable groups such as migrants.^18^ Citizens with better general IT proficiency are more informed and empowered in managing their health, leading to better health-promoting knowledge, attitudes, and behaviour.^36^ Poor IT skills and the need for guidance have been linked to non-use of personal health records and other digital health services.^37,38^

Competencies related to digital health service navigation, such as the *ability to find relevant contact information* online (rank 3) and *find relevant digital healthcare services* (rank 4), were also identified as among the most important. These competencies, often associated with the term *navigational health literacy*, are relevant, since they enable citizens to navigate healthcare systems and find the right care at the right time and place.^39^ Even in non-digital services, navigating healthcare systems can be a challenge,^39^ but the increase in digital services can add layers of complexity. To counter this, healthcare providers should support the navigation of services by making information on digital services easily accessible, helping citizens find relevant services, and understanding their benefits.^40^ According to Le et al.,^25^ healthcare navigation abilities are associated with levels of digital health literacy among adolescents.

The experts identified *using digital information sources and search systems to find information* (rank 15), *evaluating the reliability of online health information* (rank 6) and *applying it to make informed decisions in daily life* (rank 7), as well as critically evaluating *health-related discussions on social media* (rank 17) among the most important competencies. These have been the focus of much current research around digital health literacy.^21^ Their relevance has been highlighted especially during and after the COVID-19 pandemic, considering the enormous amount of false health information presented online.^41^ Individuals who are skilled at finding and using valid online health information tend to achieve better outcomes in managing their health, and are likely to adopt healthier behaviours as a result.^42^

Experts also identified *the ability to access* (rank 10) *and apply health-related information from personal health records* (rank 5) among the most important competencies. These not only offer direct access to relevant health data, but are also a key in empowering their health and collaborating with healthcare providers.^43^ However, not everyone takes or is able to take full advantage of this. Persons outside the online health record system are likely to include individuals in need of guidance in service use, in addition to those who do not have long-term illnesses and those not referred to digital healthcare services.^38^ The European Union's goal of ensuring full access to personal health records online for all citizens by 2030,^5^ including cross-border interoperability^6^ highlights broader policy efforts in this regard.

*Knowing how to apply security practices and protect privacy online* (rank 8), the *ability to share and manage personal information safely across various online platforms* (rank 11), and the *ability to provide informed consent for the digital processing and storage of personal data* (rank 12) were also identified. Such competencies form a crucial part of digital health use, enabling citizens to make informed decisions on how and where it is safe and relevant to share personal health data. However, security and trust concerns – such as fear of using available services, fear of making mistakes, and lack of confidence in service quality – remain significant barriers, especially among vulnerable groups.^18^ Notably, every third patient portal user has experienced privacy concerns,^44^ which are associated with dissatisfaction regarding the use of digital patient portals.^38^ Privacy concerns are experienced more often among older adults and those with financial difficulties.^44^

The experts also identified the *ability to make healthcare appointments electronically* (rank 9), *fill out applications related to health and wellbeing* (rank 13), *view*, *renew*, *and use electronic prescriptions* (rank 14), and *use digital citizen portals and their features* (rank 16) as among the most important competencies. As healthcare services increasingly move online, these skills will become ever more important. To take an example, the Finnish strategy for social and healthcare digitalization envisions digital services as the primary mode of interaction in the future.^45^ Individuals unable to use these services may be excluded from the full range of available solutions and their benefits.

This study also revealed competencies that were identified in the open question of the first questionnaire round but did not make it to the subsequent rounds. These skills included using real-time digital services like chat and video consultations, conducting health self-assessments and wellbeing checks, and digitally evaluating care needs. They also involved using wellbeing technologies such as smartwatches, rings, blood pressure monitors, and mobile apps, as well as skills based on artificial intelligence, like using AI-based chatbots. The importance of these competencies is likely to increase in the near future, with the technologies becoming more widespread across healthcare and health promotion.^16^ Additionally, competencies related to health advocacy were also identified in the first round but did not receive high ratings in subsequent rounds, despite being a key aspect of health promotion.^46^ These included *the ability to advocate for important issues in digital environments for oneself or others*, and *the ability to advocate for oneself in online healthcare decision-making processes.*

As Norman and Skinner note, digital health literacy ‘is not static; rather it is a process-oriented skill that evolves over time as new technologies are introduced and the personal, social and environmental contexts change’.^23^ In response to this evolving landscape, researchers have broadened the concept beyond the foundational Lily Model's focus on online health information comprehension^23^ towards dimensions like online interaction, privacy protection,^47^ active engagement and motivation to engage with digital services, feeling safe and in control, access to digital services that work^48^ to even self-managing health data and applying information.^49^ Kaihlanen et al.^24^ and Le et al.^25^ emphasise the need to expand digital health literacy research to cover competencies such as security, privacy, healthcare system navigation, self-care, online communication, and citizens’ general digital skills and readiness to engage with digital healthcare services. The findings of this study support these perspectives: citizens require a broad range of competencies to promote and maintain good health and wellbeing through digital technology. This diversity of needs should be taken into account when developing strategies to enhance digital health literacy.

Inadequate digital health literacy can limit citizens’ abilities to benefit from technology, thereby increasing digital health inequities.^12,17^ To decrease these inequities, citizens are often encouraged to improve their competencies. However, this should not be viewed solely as an individual's responsibility, but rather as a shared societal effort. Digital health literacy needs to be addressed also at organisational, commercial, technical, and political levels.^12^ To ensure equitable access, digital health resources must be accessible, user-friendly, and easy to understand – regardless of a person's skills or experience. Healthcare professionals, in turn, must be equipped to meet the challenges and opportunities posed by digitalisation and by increasingly informed and active patients.^50^ For those unable to use digital tools, alternative formats and support must be guaranteed. Governments have a key role in supporting digital health literacy, ensuring that all citizens can fully benefit from digital transformation.^51^ Nutbeam^52^ argues, digital health literacy can be developed, but it fundamentally depends on inclusive, equitable access to quality education and lifelong learning. School curricula play a central role in this regard.^53^ Moreover, any interventions – whether educational, training-based, or supportive – should be proportionate to population needs.^15^

This study was limited by cultural and geographical factors, as it included only Finnish experts. The findings may not be generalisable to countries with different healthcare systems or levels of digitalisation. The sample size was relatively small, and most participants had backgrounds in research or education and held a doctoral or equivalent degree. Their perspectives may differ from those in more practice-oriented roles. Additionally, the majority of respondents in the final round were female (87%), and it is unclear whether a more balanced gender distribution would have influenced the results.

Future research should examine digital health literacy and required competencies in diverse international contexts and incorporate the perspectives of citizens to reflect their perceived needs. Given the study's focus on competencies relevant to the next 3 years – and the rapid pace of technological change – ongoing research is essential to identify the digital health competencies needed at any given time.

## Conclusion

The findings suggest that being digitally health literate in the modern world goes beyond the ability to seek, assess, understand, and apply online health information. It also encompasses electronic identification, general IT proficiency, navigating and using digital health services, accessing personal health records, and ensuring online privacy and security. These results offer important insights into the competencies needed to meet current digital health challenges, with implications for both assessment tools and intervention strategies. As technology continues to evolve, research and evaluation methods must keep pace. Interventions aimed at improving digital health literacy should move beyond a narrow focus on information comprehension and instead address a broader and more inclusive set of competencies.
